# Clinical Research and Public Health in the *JCI*

**DOI:** 10.1172/JCI190119

**Published:** 2025-02-17

**Authors:** Elizabeth M. McNally

In this issue of the *JCI*, we are pleased to publish work from Abend et al. describing the use electronic health records (EHR) information to estimate the prevalence of autoimmune disorders in the US (1). Autoimmunity contributes to a host of diseases affecting adults and children, and the broad array of clinical diagnoses linked to autoimmunity makes it challenging to consider autoimmune diseases in aggregate. Recent estimates from the UK are enabled by having a single public health care system (2). In the US, EHR data collated from representative health care systems is well positioned to guide questions like these. Diagnostic and billing codes, laboratory values, radiographic and histopathology findings, and care-related notes can be combined to follow health outcomes over time and yield new findings on nearly every aspect of clinical and even molecular medicine. EHRs can also be exploited to iteratively implement and assess outcomes as learning health systems. As large language models better incorporate this information, it is increasingly possible to blend these data with molecular features to gain disease insight. At the population level, EHR data can highlight rapidly shifting disease trends, as it did in the COVID-19 pandemic (3).

Abend and colleagues studied health data from six large US medical systems, amassing data covering more than 100 autoimmune conditions. In total, over 10 million lives were evaluated, and more than 581,000 individuals were identified as being affected by an autoimmune conditions, for a prevalence of 4.6% of the US population. For nearly 30% of the 105 conditions, there were few-to-no patients identified, leaving 74 conditions. Females were nearly twice as likely to be diagnosed with an autoimmune disorder, with rheumatoid arthritis and psoriasis as the most common diagnoses.

The *JCI* was founded in 1924 to help the American Society of Clinical Investigation (ASCI) publish what its members considered “new” science, including defining human and animal physiology and pathophysiology. The ASCI now has a substantial component of its membership conducting investigation using large clinical datasets that have been assembled through sponsored research projects, public health efforts, and EHR data. In keeping with the Journal’s mission to meet the needs of the ASCI, we have expanded our former “Clinical Medicine” category to include both “Clinical Research and Public Health”. Reports in this category include first-in-human clinical trials, Phase 2 clinical trials, observational analyses, epidemiological studies, health disparities research, and outcomes and implementation research. Many larger human datasets are increasingly adding genomic, transcriptomic, metabolomic, and other molecular correlates. We expect the Clinical Research and Public Health manuscript category to feature papers that merge molecular data with clinical information, improve the validation of clinical data in EMRs; develop new tools to extract information, some of which have not yet been invented; and apply paradigms that have yet to be defined. The *JCI* remains keenly interested in new mechanistic insight on human disease. We recognize that this knowledge derives from many different types of research and clinical analyses, some of which will require experimental model systems and some that will not.

## Version 1 02/17/2025

Electronic publication
